# Impact of Income Inequality and Other Social Determinants on Suicide Rate in Brazil

**DOI:** 10.1371/journal.pone.0124934

**Published:** 2015-04-30

**Authors:** Daiane Borges Machado, Davide Rasella, Darci Neves dos Santos

**Affiliations:** 1 Institute of Collective Health, Federal University of Bahia—UFBA, Rua Basílio da Gama s/n, Canela, 40110–040 Salvador, Bahia, Brazil; 2 Instituto de Saúde Coletiva, Universidade Federal da Bahia, Rua Basílio da Gama s/n—Canela, 40110–040 Salvador, Bahia, Brazil; Medical University of Vienna, AUSTRIA

## Abstract

Studies about suicide worldwide have mainly focused on individual-level psychiatric risk factors. In Brazil, suicide is an important public health problem. Brazil has evidenced important socioeconomic changes over the last decades, leading to decreasing income inequality. However, the impact of income inequality on suicide rate has never been studied in the country.

**Purpose:**

To analyze whether income inequality and other social determinants are associated with suicide rate in Brazil.

**Method:**

This study used panel data from all 5,507 Brazilian municipalities from 2000 to 2011. Suicide rates were calculated by sex and standardized by age for each municipality and year. The independent variables of the regression model included the Gini Index, per capita income, percentage of individuals with up to eight years of education, urbanization, average number of residents per household, percentage of divorced people, of Catholics, Pentecostals, and Evangelicals. A multivariable negative binomial regression for panel data with fixed-effects specification was performed.

**Results:**

The Gini index was positively associated with suicide rates; the rate ratio (RR) was 1.055 (95% CI: 1.011–1.101). Of the other social determinants, income had a significant negative association with suicide rates (RR: 0.968, 95% CI: 0.948–0.988), whereas a low-level education had a positive association (RR: 1.015, 95% CI: 1.010–1.021).

**Conclusions:**

Income inequality represents a community-level risk factor for suicide rates in Brazil. The decrease in income inequality, increase in income per capita, and decrease in the percentage of individuals who did not complete basic studies may have counteracted the increase in suicides in the last decade. Other changes, such as the decrease in the mean residents per household, may have contributed to their increase. Therefore, the implementation of social policies that may improve the population’s socioeconomic conditions and reduce income inequality in Brazil, and in other low and middle-income countries, can help to reduce suicide rates.

## Introduction

Suicide is one of the main causes of death in the world and is an important public health problem. With a rate of 16 per 100,000 individuals worldwide, suicide is the cause of one million deaths per year. Some projections indicate that this rate will increase substantially over the next decades [1,2].

Brazil is among the top 10 countries in terms of deaths due to suicide [3]. It represents the 3^th^ leading cause of death due to known external causes [4] and the trend shows that this rate has continued to increase in the country over the past years [5].

Suicide is a multicausal phenomenon that results from interactions of psychological, social, biological, cultural, economic and environmental factors [1, 6]. Therefore, individual risk factors alone do not explain regional variations in rates. Data suggest that the particularities of a region at a specific period of time result in higher or lower suicide rates.

The rates of suicide increase during periods of economic crises, when unemployment and poverty rates tend to rise [7,8]. The socioeconomic context of the community may affect the health of all residents, as the characteristics of the physical environment and the availability of services vary according to socioeconomic factors [9, 10]. In addition, an individual’s socioeconomic position impacts his/her level of material living standards, exposure to environmental risk factors [10], and behavioral and psychosocial factors, such as the perception of violence, feelings of deprivation, and stress [9].

Few researches have investigated the association between suicide and inequality. Most of these studies have been done in European countries or rich countries out of Europe and did not found statistically significant association between these two factors [11,12,13,14] however two recent studies in Japan showed that the suicide rates is positively associated with income inequality [15, 16]. It must be considered that high-income countries have in general lower inequality than South America [17], and determinants of suicide may be different according to the country [16]. Therefore, to contribute with this discussion, searching for possible answers, it is very important to produce knowledge from low and middle countries, where the effect indeed can be measured. In Brazil the income inequality brings for the population unequal access to goods [18] which can promote feeling of unfair and stimulate social fragmentation. Therefore the recent period of economic transition, with decreasing the income inequality, may have impacted on suicide rates since it may have helped to improve life conditions in the Brazilian communities.

Despite being classified as the 7^th^ richest country in the world and the 2^nd^ richest in the western hemisphere in terms of nominal Gross Domestic Product, Brazil has one of the highest levels of income inequality in the world [17, 20]. This inequality has deep historic and regional roots and remains a current issue for the country. Furthermore, due to this inequality, the poor individuals in Brazil benefit less from economic growth than do those in more equal societies [18].

Recently, there have been some improvements in the country. Brazil is characterized by a fluctuating economy. Since 2003, it experienced one of the largest poverty reductions in a short period [21]. Although substantial progress has been made, income inequality remains rather high and persistent in Brazil [18] and impacts the population’s health and causes of death. In Brazil, deaths due to infectious diseases are decreasing, whereas those due to external causes are rapidly increasing [4]. In this scenario of important socioeconomic and epidemiological transitions in Brazil, the effects of income inequality and other important social determinants, such as education and income, on suicide rates have not been previously studied. In fact there is only one study that examined the effect of Income Inequality on a health outcome in Brazil, and it is not about suicide [19].

The majority of studies on suicide have been conducted in developed countries. However, most deaths by suicide occur in developing countries [6]. Therefore, increased knowledge of the suicide risk factors in specific contexts is necessary to adopt effective prevention strategies [6]. Similar to other developing countries, due to the economic fluctuations in Brazil, it is a vast laboratory to investigate the socioeconomic risk factors for suicide. Therefore, the objective of the present study was to determine whether income inequality and social determinants of health were associated with mortality due to suicide in the last decade in Brazil.

## Method

This study had a mixed ecological design and used panel data from the 5,507 Brazilian municipalities that existed over the entire period of analysis. All municipalities were examined via repeated observations over 12 years, from the year 2000 to the most recently available data in 2011. The panel dataset was balanced for all variables.

### Data Sources

The mortality data were collected from the Mortality Information System of the Brazilian Ministry of Health. The socioeconomics variables (the Gini Index, the per capita income, education, the urbanization rate, the average residents per household, the percentage of divorced people, and the percentage of Catholics, Pentecostals and Evangelicals) were obtained from the Brazilian Institute of Geography and Statistics. Because data for the socioeconomic variables were obtained from the 2000 and 2010 national census databases, we calculated the annual values from 2001–09 by linear interpolation and those for the year 2011 by linear extrapolation. The linear trend behavior was confirmed by analyses of nationwide socioeconomic surveys that were conducted every year and collected by state-level representatives [22].

### Definition of the Variables

Suicide was defined as death resulting from *intentional self-harm* according to the International Classification of Diseases, 10th revision [23], codes X60 to X84. The outcome variable, suicide rates were calculated at the municipality level and standardized by age (for every five years) with a direct method using the WHO population as model for individuals over 10 years old. Suicide was analyzed by the overall rate and by sex for each municipality and year of analysis. Although suicide deaths can be underestimated due to stigmatization and social taboo, a review found that this underestimation is not sufficient to bias research results [24].

One of the main independent variable was the Gini Index, which measures the degree of concentration of the distribution of household income per capita in a given geographical area. It was calculated by measuring the area between the Lorenz curve and a hypothetical line of absolute equality, expressed as a percentage of the maximum area under the line. It varies on a scale from 0 to 100, with 0 representing the most equal income levels and 100 representing the most unequal income levels [17]. The control variables included the monthly per capita income BR$, percentage of individuals who did not complete basic studies, measured by proportion of individuals over 10 years old with up to eight years of education, minimum period required in Brazil, the urbanization rate, as measured by the percentage of individuals living in urban areas, the mean number of residents per household, and the percent of individuals who self-declared as Catholic, Pentecostal, or Evangelical. Considering that the average change of Gini Index and per capita income during the study period we scaled the units of these two variables; Gini Index of 54 was introduced as 5.4 and per capita income of 100 reals were introduced as 1, for example.

### Statistical Analysis

Three regression models were performed. In **model 1**, the independent variables, which represented the main social determinants of suicide rates, were as follows: the Gini Index, the per capita income, education, the urbanization rate, and the mean residents per household. In **model 2**, only demographic variables, i.e., the percentage of individuals who self-declared as divorced, Catholic, Pentecostal, and Evangelical, were tested. Finally, **model 3** controlled for all variables in the two previous models. A time variable (time-specific effect) used as continued variable was introduced into the models to control for the national-level policy changes or secular trends that could affect all of the municipalities [25]. The time variable was used as years from 2000 to 2011. Also, a sensitive analysis using the time as a dummy variable was performed resulting in very similar outcomes.

A multivariate regression analysis was performed using a negative binomial (NB) regression for panel data with fixed-effects (FE) specification in the three models. NB regression models are used when the outcome variable is considered count data and the Poisson model assumption that the mean is equal to the variance does not hold true, typically because the data are over-dispersed [26].

The estimated regression model was: ln(Y_it_) = α_i_ + β1Gini_it_ + βnXn_it_ + γ_t_ + u_it,_ where Y_it_ was the suicide rate for the municipality i in year t, α_i_ was the fixed effect for the municipality i that captured all unobserved time-invariant factors, Gini_it_ was the Gini Index for the municipality i in the year t, Xn_it_ was the value of each n covariate of the model, including all socioeconomic determinants, in the municipality i in the year t, γ_t_ was the time-specific effect, and u_it_ was the error.

An FE specification was chosen based on the Hausman test and the argument that the time-invariant term could control for unobserved characteristics of the municipality, such as geographical, historical, socio-cultural, or socioeconomic characteristics that did not change during the study period and could be correlated with the independent variables of the model.

A series of sensitivity tests was performed to analyze the robustness of the results. We fitted models with different specifications from the same dataset, including Poisson regressions with robust SEs. The Akaike's Information Criterion (AIC) and the Bayesian Information Criterion (BIC) were used to establish which model best fit the data [26]. Negative regression models with FE specification using only the data of 2000 and 2010 were performed to confirm that interpolation values had not influenced the results. Difference-in-difference models were performed as sensitivity tests. None of the regression models with different specifications provided estimates that contradicted the study results. The conditional FE NB regression models were considered the most appropriate for the analysis. All statistical analyses were conducted using Stata (v.12).

## Results

Table 1 shows the mean values and the percentage changes of the dependent and independent variables. The suicide rates increased overall for both men and women. The socioeconomic conditions improved with a decrease in the Gini Index and the percentage of individuals who did not complete basic studies and an increase in the per capita income from 2000 to 2011. There was an increase in urbanization and the percentage of individuals who were divorced, Evangelicals, and Pentecostals. By contrast, there was a decrease in the average number of residents per household and the percentage of Catholics.

**Table 1:** Mean values and SD of the selected variables for the Brazilian municipalities (n = 5,507).

	Mean 2000	SD 2000	Mean 2011	SD 2011	Percentage of Change
Suicide rate*	6.49	13.40	7.71	12.79	18.80
Suicide rate among men*	10.47	23.26	12.48	22.64	19.20
Suicide rate among women*	2.45	12.08	2.92	10.04	19.18
Gini Index	55.44	6.84	49.81	6.97	-10.16
Per capita income BR$ (monthly)	336.96	199.87	496.87	245.07	47.46
Percentage of individuals who did not complete basic studies	83.88	7.25	61.18	10.21	-27.06
Urbanization rate	58.75	23.34	64.64	21.84	10.03
Mean number of residents per household	3.93	0.52	3.34	0.43	-15.01
Percentage of individuals who were divorced	2.35	1.53	3.75	1.92	59.57
Percentage of Catholics	81.91	11.65	75.04	13.61	-8.39
Percentage of Pentecostals	8.21	5.58	11.26	6.62	37.15
Percentage of Evangelicals	11.95	8.39	17.54	9.51	46.78

Abbreviation: SD = Standard Deviation

*Rate standardized

**Fig 1:**
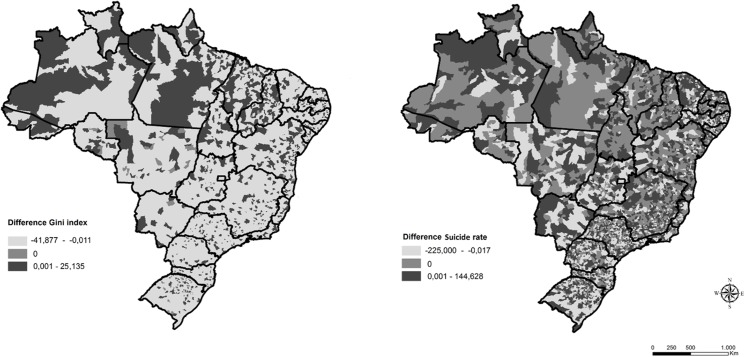
Gini Index and Suicide Rate comparison in Brazil from 2000 to 2011.

The suicide rate increased in Brazil during the study period. However, in some municipalities, the rate did not change or decreased, as shown in Fig 1. The changes in income inequality also varied from 2000 to 2011 in the municipalities under study. Income inequality decreased more in the south and center-west regions of Brazil and suicide rates followed the same tendency in those areas.

As shown in Table 2, in the regression model 1, income inequality was positively associated with suicide rates. This positive association remained statistically significant after controlling for the other important covariates, as shown in models 2 and 3. The per capita income was negatively associated with suicide, whereas the percentage of individuals who did not complete basic studies was positively associated with suicide in both models. The urbanization rate, the average number of residents per household, and the percentage of Pentecostals were negatively associated with suicide, whereas the percentage of Evangelicals was positively associated with suicide.

**Table 2:** Fixed effect regression models for adjusted associations between suicide rate and the Gini Index for the Brazilian Municipalities, 2000–2011.

Variable	Model 1		Model 2		Model 3	
	RR	(95% CI)	RR	(95% CI)	RR	(95% CI)
Gini Index	1.052	(1.010–1.096)	-	-	1.055	(1.011–1.101)
Per capita income BR$ (monthly)	0.975	(0.956–0.994)	-	-	0.968	(0.948–0.988)
Percentage of individuals who did not complete basic studies	1.016	(1.011–1.021)	-	-	1.015	(1.010–1.021)
Urbanization rate	0.992	(0.989–0.995)	-	-	0.994	(0.991–0.997)
Mean number of residents per household	0.323	(0.291–0.359)	-	-	0.323	(0.291–0.359)
Percentage of individuals who were divorced	-	-	0.962	(0.937–0.987)	0.992	(0.965–1.020)
Percentage of Catholics	-	-	1.023	(1.016–1.030)	1.007	(1.000–1.014)
Percentage of Pentecostals	-	-	0.988	(0.979–0.996)	0.974	(0.965–0.983)
Percentage of Evangelicals	-	-	1.020	(1.010–1.029)	1.016	(1.006–1.027)
Time(Year)	1.002	(0.990–1.013)	1.023	(1.018–1.029)	1.004	(0.992–1.016)
Number of observations	62484		62484		62484	
Number of municipalities	5207		5207		5207	

Abbreviations: CI = Confidence Interval; RR = Rate Ratio

The association between some of the independent variables and suicide rates varied according to gender (Table 3). This stratification showed that inequality was positively associated with suicide for both men and women. The percentage of individuals who did not complete basic studies also was negatively associated for both genders, but the percentage of individuals who were divorced was negatively associated with suicide only among women.

**Table 3:** Fixed effect regression models for adjusted associations between suicide rate and the Gini Index for the Brazilian Municipalities by gender, 2000–2011.

Variable	Male analysis model		Female analysis model	
	RR	(95% CI)	RR	(95% CI)
Gini Index	1.060	(1.012–1.111)	1.056	(0.964–1.157)
Per capita income BR$ (monthly)	0.960	(0.939–0.982)	1.000	(0.958–1.044)
Percentage of individuals who did not complete basic studies	1.012	(1.006–1.018)	1.023	(1.012–1.035)
Urbanization rate	0.995	(0.992–0.998)	0.990	(0.983–0.997)
Mean number of residents per household	0.304	(0.271–0.340)	0.441	(0.346–0.561)
Percentage of individuals who were divorced	0.984	(0.955–1.014)	1.064	(1.001–1.130)
Percentage of Catholics	1.007	(0.999–1.014)	1.003	(0.987–1.018)
Percentage of Pentecostals	0.975	(0.965–0.985)	0.973	(0.953–0.994)
Percentage of Evangelicals	1.016	(1.006–1.028)	1.019	(0.996–1.042)
Time(Year)	0.996	(0.983–1.008)	1.021	(0.995–1.048)
Number of observations	60972		44184	
Number of municipalities	5081		3682	

Abbreviations: CI = Confidence Interval; RR = Rate Ratio

## Discussion

The present study shows that income inequality is an important determinant of suicide in Brazil. Each 10-point decrease in the Gini Index would result in a 5.5% decrease in the suicide rate. According to this decrease, 538 individuals less would die each year. In the areas where the Gini Index had the greatest decrease, the south and center-west regions, the rate of suicide also decreased. Although suicide rate continues to increase in Brazil, this increase has weakened over the last few years. This effect may be due to decreases in inequality and the percentage of individuals who did not complete basic studies and increases in income. However, other factors, such as decreases in the mean number of residents per household, may contribute to the overall increase in suicide rate.

Most studies that found a significant association between socioeconomic factors and suicide rates showed a positive association between suicide rates and deprivation, low occupation-based social class, the percentage of unemployed individuals, and a low education level [27]. These results suggest that deprivation increases the risk of suicide. This association was shown in studies that were conducted in Asia, North America, Australia, and New Zealand [27]. The smaller associations in studies that were conducted in European countries may be explained by the impact of measures to promote social welfare in these areas, as such measures can mitigate the negative effects of deprivation. The mechanisms of this association in Latin America are unknown [27]. Considering that the reasons that lead individuals to commit suicide in richer and poorer countries may be distinct, as the standard of living and the problems that individuals face may be distinct, it is important to investigate these factors in developing countries.

In the current study, income inequality was positively associated with suicide rate in both genders in Brazil. The confidence interval changed among women after stratification. However, this change presumably occurred due to the limited number of observations, as the rate ration (RR) of women did not change (RR: 1.056) in Table 3 from model 3 (RR: 1.055) in Table 2, in which both genders were included. One possible explanation for these results is that income inequality increases social fragmentation, which is a risk factor for suicidal behavior [27]. As suicidal behavior can be explained as a process that results from the movement of frustration-aggression., individuals who face high levels of economic frustration, when comparing themselves with individuals living in better situations, are at a greater risk of committing an act of aggression against themselves or others than are those living in better situations [28].

Also, economic hardship can impact on suicidal behavior through psychological aspects such as depression caused by family economic instability and feeling of hopelessness, experience of persistent economic stress, anxiety for not get a job and impulsiveness [2]. The psychological distress can reach a point that feels unbearable and lead people to seek escape via suicide [2]. Therefore, intervention that focuses on more distal risk factors such as socioeconomic factors will possibly also impact on Psychological and Psychiatric proximal factors. An article review shows that prevention strategies focusing in particular socioeconomic strata will potentially have the same population-level effects of psychiatric risk factors in the prevention and control of suicide [29].

Along these lines, inequality can become a way that promotes contextual economic deprivation increasing the risk of suicide in the community. In Brazil, income inequality makes the poorer population benefits less from economic goods than does the richer population [18]. It makes interesting to investigate the income inequality in this country as it is a place where inequality really impact on people life in many sectors, promoting different access to health care and access to different levels of education for example. Thus, all these aspects can impact on people shortening the chances of poorer improve socioeconomically and therefore can contribute to feeling of hopeless.

Inequality can also impact individuals by reinforcing feelings of hopelessness and impossibility when they note that individuals around them share the same difficulties but others face a much better economic situation [30]. Differently of developed countries, in Brazil the shock of these two realities can be confronted very often, as very poor people, mainly in urban areas, can live in the same neighborhood of rich people. However, dealing with very precarious living conditions while they live in “favelas”.

Inequality can bring a sense of injustice, promoting feelings of revolt and personal bankruptcy for not achieve economic success [24,31]. Therefore, the implementation of social policies in Brazil promoting fair and adequate socioeconomic conditions has contributed to their inhabitants to manage their life issues without resorting to suicide.

The per capita income was found to be a protective factor against suicide in Brazil. This effect may occur because poverty increases the propensity for suicide due to greater exposure to suicidogenic factors, such as stress caused by financial problems, family instability, physical illness, alcoholism, alienation at work, a greater risk of being a victim of violence, and mental health problems [24]. Groups that experience economic strain, such as the poor and unemployed, can reach a situation in which the living concerns outweigh the fear of death. At that point, the costs/benefits equation can increase the suicide risk when the balance tends more toward the costs [24]. Studies have shown that regions with better socioeconomic situations have lower suicide rates for both men and women [28]. When the economy is expanding, individuals tend to experience less stress and depression [31]. The current study found that although Brazil is economically growing, this growth is not occurring equally across the country. This result may explain why suicide rates decreased in areas where the improvements occurred faster and increased in areas where the socioeconomic conditions worsened during the period of analysis.

The literature shows that education, unemployment, family income, and marital status define individuals’ economic and social positions, which provide distinct levels of worry and stress [30]. In addition, social status is experienced according to the local culture. The meaning shared by members of the group varies and can cause feelings of dissatisfaction and frustration, which can then cause psychological distress.

The percentage of individuals who did not complete basic studies was identified as a risk factor for suicide. Previous studies have found the same association [30,32]. Specifically, an individual’s education level can impact his/her self-evaluation, thereby influencing his/her self-esteem and interactions with others. Studies have shown that low self-esteem can lead to suicidal tendencies [33]. In addition, the regions of Brazil with the most concentrated percentage of individuals who did not complete basics studies also suffer from higher poverty levels. Therefore, in these regions, individuals are more exposed to difficult living conditions.

The present results show that urbanization is a protective factor against suicide in Brazil. International studies have shown mixed results [34,35]. However, it is important to consider that the urbanization patterns in developed countries are different from that in developing countries [36], such as Brazil. The results presented in the current investigation are in accordance with previous results. Overall, areas with higher urbanization rates also have higher per capita income, lesser income inequality, and greater years of education. Likewise, living in an urban area in Brazil may provide more opportunities to access healthcare in general and mental healthcare services.

In Brazil, the “psychiatric reformation” that began in 1978 reformulated the mental health system [37]. In this context, the CAPS-Psychosocial Care Center was developed to provide mental health assistance in communities and, thus, avoid hospitalizations. The first CAPS were created in 1987 [37]. Since then, the number of CAPS has steadily increased while the number of psychiatric hospitals has steadily decreased. Such assistance could help to prevent suicide across the country; however, only 21% of the municipalities in Brazil had a CAPS-Psychosocial Care Center in 2010 and none received specific preparation for suicide prevention.

The average number of residents in the household was a protective factor against suicide in the present study. A decrease in the average number of individuals in the household increased the suicide rate. Together with marital status, this factor may be a proxy for social integration, which can protect against suicide [31]. Personal relationships can provide the motivation to live. In addition, proximity to other individuals increases one’s likelihood of being referred to mental health care when needed. Previous studies have shown a positive association between the percentage of individuals who are divorced and the suicide rate [31]. The present study found the same results among women, what can be explained by familiar attachment, which is a protective factor against suicide [31]. Suicide rate is significantly higher among individuals who are single [32, 38,39] and those who live alone with no relatives in close proximity [6]. Bonding may also increase a sense of belonging and the motivation to live.

The impact of religion on suicide has been largely studied worldwide. The current study found no significant association between suicide rate and the percentage of individuals who self-declared as Catholic. This result can be explained by the traditional Catholic background in Brazil. It is common for individuals to self-declare as Catholic based on the family’s background even when they have not practiced the religion. However, a significant negative association was found between the proportion of Pentecostals and the suicide rate, confirming the results of a previous study [40]. Religion may protect against suicide due to its views concerning the suicide act. In addition, religion may help to promote community integration and, thereby, decrease the feeling of loneliness, which is a risk factor for suicide [24].

### Limitations and Strengths of the Study

One of the main limitations of this study is the impossibility to exactly determine the exposure of those individuals who experienced the outcome because information is only available at an aggregate level. However, considering the data sources and the characteristics of the outcome (e.g., suicide is a relatively rare event in Brazil), only an ecological approach allows for a nationwide longitudinal analysis in a developing big country such is Brazil. Also, it has to be considered that income inequality and other contextual determinants of suicide can only be measured at the aggregate level. However, the use of the municipality, a smaller unit of analysis than that used in previously cited studies, and the reduced variability in the variable values in each municipality, contribute to the minimization of this bias.

Therefore, ecological fallacy is less plausible in our study firstly because the vast majority of Brazilian municipalities have reduced dimensions (90% of municipalities have less than 50,000 inhabitants) and secondly because they are homogeneous in terms of our variables characteristics. As discussed in theoretical papers on ecologic studies, this reduces the possibility of ecological fallacy [41,42].

Another concern could be the completeness of the data. However, a recent article has showed that almost 80% of the Brazilian population lives in areas with satisfactory levels of death information since the beginning of the last decade (2000–2010) [43]. In addition, data on socioeconomic determinants were collected by the national census, which has a recognized high quality standard [44].

The main strength of the current study is the use of panel data rather than traditional cross-sectional data. Longitudinal data allow for the evaluation of the influence of social contextual features over time and provide stronger evidence for causal inferences. Moreover, the use of a nationwide analysis of all municipalities assures the generalizability of the results to all Brazil. However, due to different socioeconomic and cultural patterns, the findings of this study are not necessarily generalizable to all developing countries, but only to these more similar, from a socioeconomic and cultural point of view to Brazil, such as Latin-American countries.

### Conclusions

Suicide is an individual act; however, it is influenced by and impacts the collective society. The current study suggests that in addition to psychiatric individual-level factors, socioeconomic determinants are risk factors for suicide rate. High income inequality as it can increase the hardships of the poor as well as low income per capita and high proportion of people who did complete basic studies represents important risk factors for suicide. Therefore, the implementation of social policies which may improve the population’s socioeconomic conditions and reduce income inequality in Brazil, and in other low and middle-income countries, is advisable to reduce suicide rate. Regarding the mental health care policies, the focus on the socioeconomically vulnerable could help to decrease the suicide rates.

## References

[1] WHO, Suicide Prevention and special programs. Available: http://www.who.int/mental_health/prevention/suicide/suicideprevent/en/. Accessed 10 November 2013.

[2] Nock MK, Borges G, Bromet EJ, Cha CB, Kessler RC, Lee S. Suicide and suicide behavior. Epidemiol Rev 2008; 30:133–154 10.1093/epirev/mxn002 18653727 PMC2576496

[3] de Mello-Santos C, Bertolote JM, Wang Y-P. Epidemiology of suicide in Brazil (1980–2000): characterization of age and gender rates of suicide. Rev Bras Psiquiatr 2005; 27:131–134 15962138 10.1590/s1516-44462005000200011

[4] Reichenheim ME, de Souza ER, Moraes CL, de Mello-Jorge MHP, da Silva CMFP, de Souza Minayo MC. Violence and injuries in Brazil: the effect, progress made, and challenges ahead. Lancet 2011; 377:1962–1975 10.1016/S0140-6736(11)60053-6 21561649

[5] Prevenção do suicídio: manual dirigido a profissionais das equipes de saúde mental, Ministério da Saúde, 2005 Available: http://bvsms.saude.gov.br/bvs/publicacoes/manual_editoracao.pdf. Accessed 3 June 2013.

[6] Hawton K, Heeringen KV. Suicide. Lancet 2009; 373:1372–1381 10.1016/S0140-6736(09)60372-X 19376453

[7] Stuckler D, Basu S, Suhrcke M, Coutts A, McKee M. The public health effect of economic crises and alternative policy responses in Europe: an empirical analysis. Lancet 2009; 374:315–323 10.1016/S0140-6736(09)61124-7 19589588

[8] Uutela A. Economic crisis and mental health. Curr Opin Psychiatry 2010; 8:127–130 10.1097/YCO.0b013e328336657d 20087188

[9] Robert SA. Socioeconomic position and health: the independent contribution of community socioeconomic context. Annu Rev Sociol 1999; 25:489–516

[10] Subramanian PB, Kawachi I. The macroeconomic determinants of health. Annu Rev Public Health 2002; 23:287–302 11910064 10.1146/annurev.publhealth.23.100901.140540

[11] Lynch J, Smith GD, Hillemeier M, Shaw M, Raghunathan T, Kaplan G. Income inequality, the psychosocial environment, and health: Comparisons of wealthy nations, The Lancet 358: 194–200, 2001 11476836 10.1016/S0140-6736(01)05407-1

[12] Andres AR. Income inequality, unemployment, and suicide: A panel data analysis of 15 European countries, Applied Economics 37: 439–51, 2005

[13] Leigh A, Jencks C. Inequality and mortality: Long-run evidence from a panel of countries, Journal of Health Economics 26: 1–24, 2007 16963138 10.1016/j.jhealeco.2006.07.003

[14] Minoiu C, Andres AR. The effect of public spending on suicide: Evidence from U.S. state data, Journal of Socio-Economics 37: 237–61, 2008

[15] Chen J, Choi YJ, Sawada Y. How is suicide different in Japan?, Japan and the World Economy 21: 140–50, 2009

[16] Kazuyuki I. Income inequality and the suicide rate in Japan: Evidence from cointegration and LA-VAR. Journal of Applied Economics, Universidad del CEMA 2010; 0: 113–133

[17] GINI index. The World Bank, 2013. Available: http://data.worldbank.org/indicator/SI.POV.GINI/countries/%201W?display=default. Accessed 20 November 2013.

[18] Velez CE, de Barros RP, Ferreira F, Elbers C, Lanjouw JO, Lanjouw P, et al Inequality and economic development in Brazil World Bank country study. Washington, DC, The World Bank, 2004

[19] Rasella D, Aquino R, Barreto ML. Impact of income inequality on life expectancy in a highly unequal developing country: the case of Brazil Journal of epidemiology and community health 67 (8), 661–666, 2013 10.1136/jech-2012-201426 23637304

[20] GDP ranking. The World Bank, 2013. Available: http://data.worldbank.org/data-catalog/GDP-ranking-table. Accessed 19 November 2013.

[21] IPEA. A Década Inclusiva (2001–2011): Desigualdade, Pobreza e Políticas de Renda, Comunicados do IPEA, 2012; N° 155.

[22] IBGE website. Pesquisa nacional por amostra de domicílios (PNAD, População). Instituto Brasileiro de Geografia e Estatística. Available: http://www.ibge.gov.br/home/estatistica/pesquisas/pesquisa_resultados.php?id_pesquisa=40. Accessed 6 Jun 2013.

[23] International classification of diseases: ICD-10 Geneva, World Health Organization, 1992

[24] Stack S. Suicide: A 15-year review of the sociological literature part I: cultural and economic factors. Suicide Life-Threat 2000; 30:145–162 10888055

[25] Wooldridge JM. Introductory econometrics, a modern approach, 3rd edn. Cinicinnati, South-Western College Publishers, 2005

[26] Hilbe JM. Negative binomial regression Cambridge, UK, Cambridge University Press, 2007

[27] Rehkopf DH, Buka SL. The association between suicide and the socio-economic characteristics of geographical areas: a systematic review. Psychol Med 2006; 36:145–157. 16420711 10.1017/S003329170500588X

[28] Ying YH, Chang K. A study of suicide and socioeconomic factors. Suicide Life-Threat 2009; 39:214–226 10.1521/suli.2009.39.2.214 19527162

[29] Li Z, Page A, Martin G, Taylor R. Attributable risk Of psychiatric and socio-economic factors for suicide from individual-level, population-based studies: A systematic review. Social Science and Medicine 2011; 72 4: 608–616 10.1016/j.socscimed.2010.11.008 21211874

[30] Desaulniers J, Daigle MS. Inter-regional variations in men's attitudes, suicide rates and sociodemographics in Quebec (Canada). Soc Psychiatry Psychiatr Epidemiol 2008; 43:445–453 10.1007/s00127-008-0340-2 18404236

[31] Kõlves K, Milner A, Värnik P. Suicide rates and socioeconomic factors in Eastern European countries after the collapse of the Soviet Union: trends between 1990 and 2008. Sociol Health Illn 2013; 35:956–970 10.1111/1467-9566.12011 23398609

[32] Stevović LI, Jašović-Gašić M, Vuković O, Peković M, Terzić N. Gender differences in relation to suicides committed in the capital of Montenegro (Podgorica) in the period 2000–2006. Psychiatr Danub 2011; 23:45–52 21448096

[33] Mann M, Hosman CMH, Schaalma HP, de Vries NK. Self-esteem in a broad spectrum approach for mental health promotion. Health Educ Res 2004; 19:357–372 15199011 10.1093/her/cyg041

[34] Otsu A, Araki S, Sakai R, Yokoyama K, Scott Voorhees A. Effects of urbanization, economic development, and migration of workers on suicide mortality in Japan. Soc Sci Med 2004; 58:1137–1146 14723908 10.1016/s0277-9536(03)00285-5

[35] Shah A. A cross-national study of the relationship between elderly suicide rates and urbanization. Suicide Life-Threat 2008; 38:714–719 10.1521/suli.2008.38.6.714 19152302

[36] Puga D. Urbanisation patterns: European vs less developed countries, LSE Research Online Documents on Economics, London School of Economics and Political Science, LSE Library 1996; 20656: 1–28

[37] Reforma psiquiátrica e política de saúde mental no Brasil Brasília, Ministério da Saúde, 2005

[38] Sarma K, Kola S. Firearm suicide decedents in the Republic of Ireland, 1980–2005. Public Health 2010; 124:278–283 10.1016/j.puhe.2010.02.018 20363005

[39] Kleina SD, Bischoffa C, Schweitzerb W. Suicides in the Canton of Zurich (Switzerland). Swiss Med Wkly 2010; 140:w13102 10.4414/smw.2010.13102 22052542

[40] Filho JGB, Werneck GL, de Almeida RLF, de Oliveira MIV, Magalhães FB . Estudo ecológico sobre os possíveis determinantes socioeconômicos, demográficos e fisiográficos do suicídio no Estado do Rio de Janeiro, Brasil, 1998–2002. Cad Saúde Pública 2012; 28:833–844 10.1590/s0102-311x2012000500003 22641507

[41] Haynes R, Lovett A, Reading R, Langford I, Gale S. Use of homogeneous social areas for ecological analyses: A study of accident rates in pre-school children. European Journal of Public Health 1999; 9: 218–222

[42] Wakefield J. Ecologic Studies Revisited. Annu. Rev. Public Health 2008; 29: 75–90 17914933 10.1146/annurev.publhealth.29.020907.090821

[43] Szwarcwald CL. Strategies for improving the monitoring of vital events in Brazil. Int J Epidemiol 2008; 37: 738–44. 10.1093/ije/dyn130 18653509

[44] Instituto Brasileiro de Geografia e Estatística (IBGE). Available: http://www.ibge.gov.br/home/. Accessed 15 November 2013.

